# Supplementary material for: General object-based features account for letter perception
Source: PLoS Comput Biol. 2022 Sep 26;18(9):e1010522. doi: 10.1371/journal.pcbi.1010522 (PMC9536565; doi:10.1371/journal.pcbi.1010522)
Supplement: S1 Text — Additional details provided for the decoding analyses from object features, model comparisons, and how we related behavioral measurements to neural network feature spaces (PDF) [file pcbi.1010522.s001.pdf]

Supplementary Text for

General object-based features account for letter perception

Daniel Janini, Chris Hamblin, Arturo Deza, Talia Konkle

Corresponding author: Daniel Janini
Email: daniel_janini@g.harvard.edu

S1: Decoding analyses from object-based features.

To test the plausibility of recycling object-based features for letter perception, we conducted decoding analyses. First, we tested whether object-based features support the linear classification of letter identity across font and size variation. For each layer of AlexNet trained on ImageNet, we measured responses to images of the 26 lowercase letters across 550 fonts and 5 sizes ranging from 45 pixels to 224 pixels. We trained linear classifiers on 80% of the fonts and sizes, then we tested the classifier on letter images from the left-out size and fonts. While classification accuracy was above chance for all layers (all p<10^-4), it reached a plateau beginning with Layer 3, with decoding accuracies ranging from 95-97% between Layers 3-7 (chance-level: 50%). Thus, we found that features of object-trained networks with mid-to-high level layer depth best distinguished between letter categories, complementing our findings that these feature spaces best correlated with the visual similarity of letters.

Next, we tested whether the letter-preferring subspace of the object-based feature space especially carried information on letter identity. For each layer, we trained and tested classifiers on the features which preferentially responded to letters over objects. As a point of comparison, we also trained classifiers on randomly selected sets of features matching the size of the letter-preferring subspace. Decoding accuracy was higher in the letter-preferring subspace than the randomly selected subspace in Layers 2-5 and 7 (all p<10^-4), though decoding accuracy was a bit higher in the randomly selected subspace for Layer 6 (p<10^-4). Thus, in most layers of the object-trained network, the letter-preferring feature subspace better distinguished between letter categories. These results are suggestive that read-out mechanisms which preferentially operate over a letter-preferring subspace of an object-based feature space will more accurately represent the identity of letters across font and size variation. However, to fully investigate how letter identities are read out from visual features, one would need to more fully compare multiple models of read out mechanisms, linking the algorithms of classification to neural mechanisms of read out operating over the visual system.

S3: Comparison of letter perception to model from Testolin et al. (2017).
In addition to our specialized letter models, we considered a previously published model from Testolin et al. (2017), which learns specialized letter features which operate over domain-general low-level features. This model includes four layers: 1) a layer which performs a whitening algorithm over the image input, 2) a layer of primitive domain-general features acquired from unsupervised learning over natural scene images, 3) a layer of specialized letter features acquired from unsupervised learning over letter images, 4) a linear read out layer which classifies letter identity. The original model from Testolin et al. (2017) was trained on an image set of uppercase letters, so to compare their model to our behavior data, we trained it on our lowercase GoogleFonts database. The model was trained according to specifications from the original paper and code available on their OSF repo (https://osf.io/s6ytk/). We calculated the RDMs from the primitive domain-general features and the specialized letter features of their model and compared them to our two behavioral experiments. The RDM from these specialized letter features exhibited moderate correlations with both of our behaviorally measured RDMs ($\rho = 0.45$, $\rho = 0.39$ for visual search and categorization, respectively). However, Layers 3-7 of object-trained AlexNet exhibited higher correlations with both behaviors (all $p \leq 0.003$, bootstrap resampling of letter pairs). Thus, the mid-to-late level features of object-trained AlexNet better accounted for human perception of letters than an unsupervised architecture recycling low-level general features.

Relating behavioral measurements to neural network feature spaces.

Comparing measurements from human behavior with measurements of representational similarity in a neural network requires making certain assumptions about the mechanisms of behavior during the experimental task. When a participant distinguishes between stimuli in a visual search or categorization task, we assume that a read-out mechanism operating over a visual feature space is supporting the behavior. We begin with the simple assumption that no matter the read-out mechanism, representations that are closer together in the feature space will be harder to distinguish than representations that are farther apart. For the visual search experiment, participants needed to find the odd-one-out without knowing the letter identities of the target and distractor before each trial. We propose they accomplished this by computing the visual similarity between each pair of items that appeared next to each other on the screen – more specifically by taking the Euclidean distance between their features. The distractors were always the same item, so they did not differ in their features. In this context, the task of identifying the target is synonymous with identifying the location at which the similarity between an item and its neighbor is not zero. When the target shared features with the distractors, the measure of its dissimilarity to its neighboring distractors would be small, so it would take more time to locate. For the categorization experiment, we propose a similar behavioral mechanism: the participant computed the Euclidean distance between the features present in the item on the screen and the average features present in the letter identity they were comparing it to. The more the letter on the screen shared features with the letter identity they were comparing it to, the longer it would take for the participant to know that the feature distance between the two stimuli was greater than 0, and to respond that they were different. Under these assumptions computing the Spearman correlation between each of our model RDMs and our behaviorally measured RDMs is appropriate.

Supplementary Text References

Testolin A, Stoianov I, Zorzi M. Letter perception emerges from unsupervised deep learning and recycling of natural image features. Nat Hum Behav. 2017 Sep;1(9):657–64.
