# Supplementary figures and images for: General object-based features account for letter perception
Source: PLoS Comput Biol. 2022 Sep 26;18(9):e1010522. doi: 10.1371/journal.pcbi.1010522 (PMC9536565; doi:10.1371/journal.pcbi.1010522)

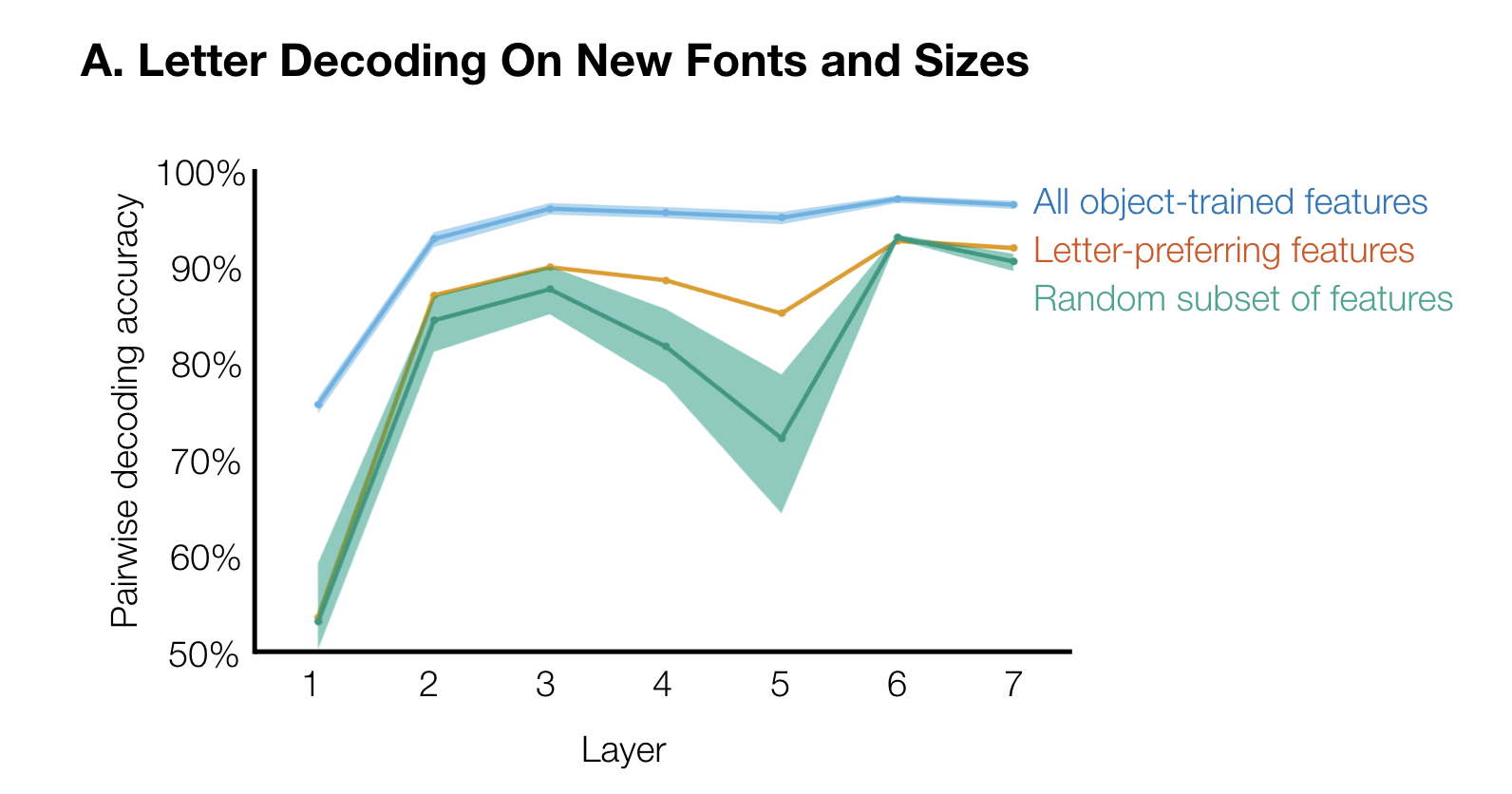

Supplement: S1 Fig — A. Linear support vector machines were trained to categorize letters across font and size variation from the features of AlexNet trained on ImageNet. Three subsets of features are compared: 1) all the features from each layer, 2) only the features from each layer which preferentially responded to letters over object images, 3) a random subset of features matching the number of letter-preferring features. Classifiers were trained on random sets of letter fonts and sizes, then tested on left out fonts and sizes. The shaded areas indicate the 95% confidence interval across random testing/training splits. For the random subset of features, the confidence interval also includes variance introduced by the random selection of features during each instance of classifier training and testing. (TIFF) [file pcbi.1010522.s002.tiff]

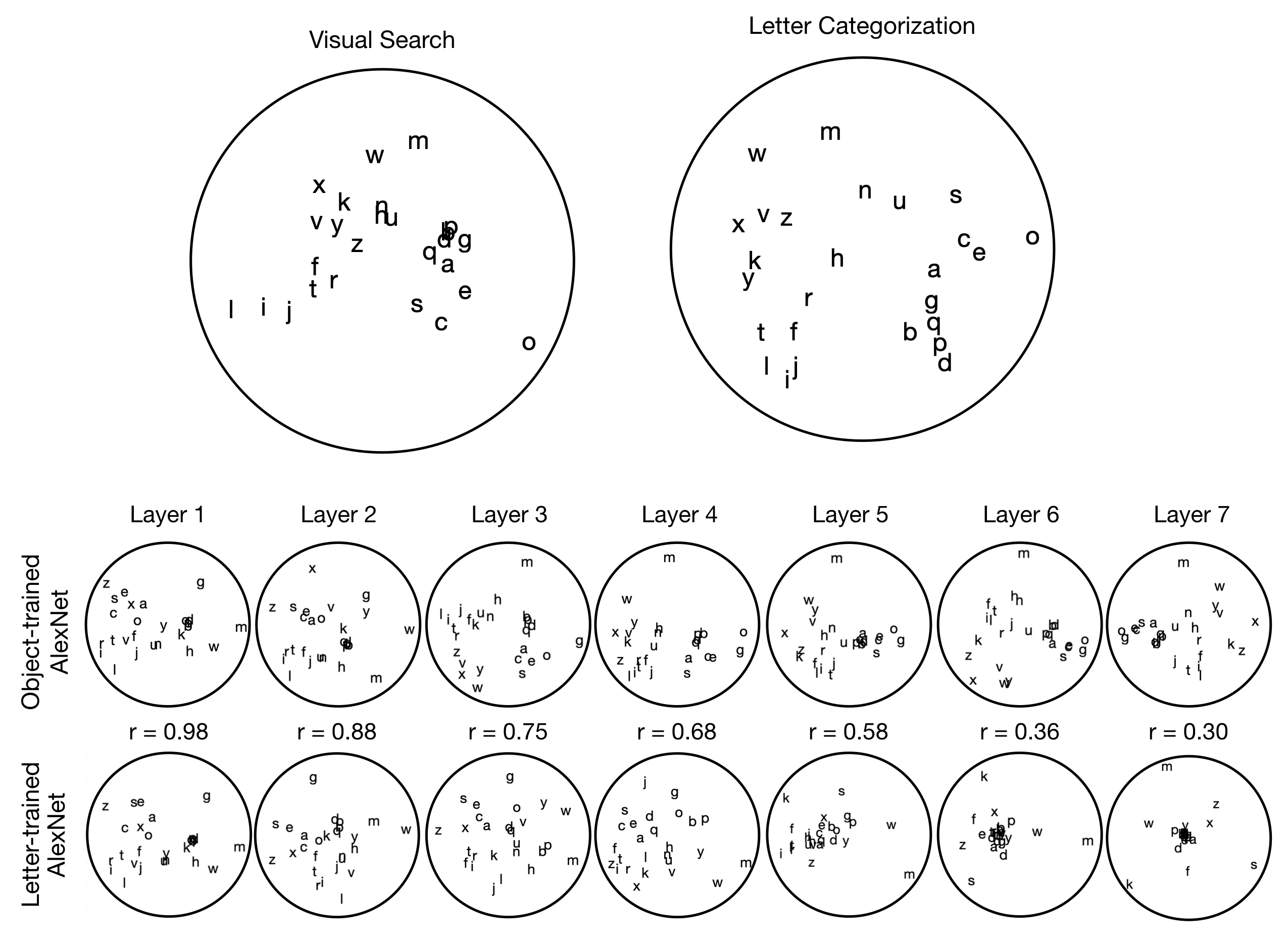

Supplement: S2 Fig — Multidimensional scaling was used to project 26x26 RDMs onto two dimensions. Distance between letters illustrates their similarity as measured during visual search and letter categorization (above). Layer-wise MDS plots for object-trained AlexNet (middle) and letter-trained AlexNet (bottom) are also illustrated. Please note that reducing the dimensionality of neural network feature spaces to two dimensions obscures a lot of meaningful variance, and these visualizations are only for exploratory inspection. (TIFF) [file pcbi.1010522.s003.tiff]

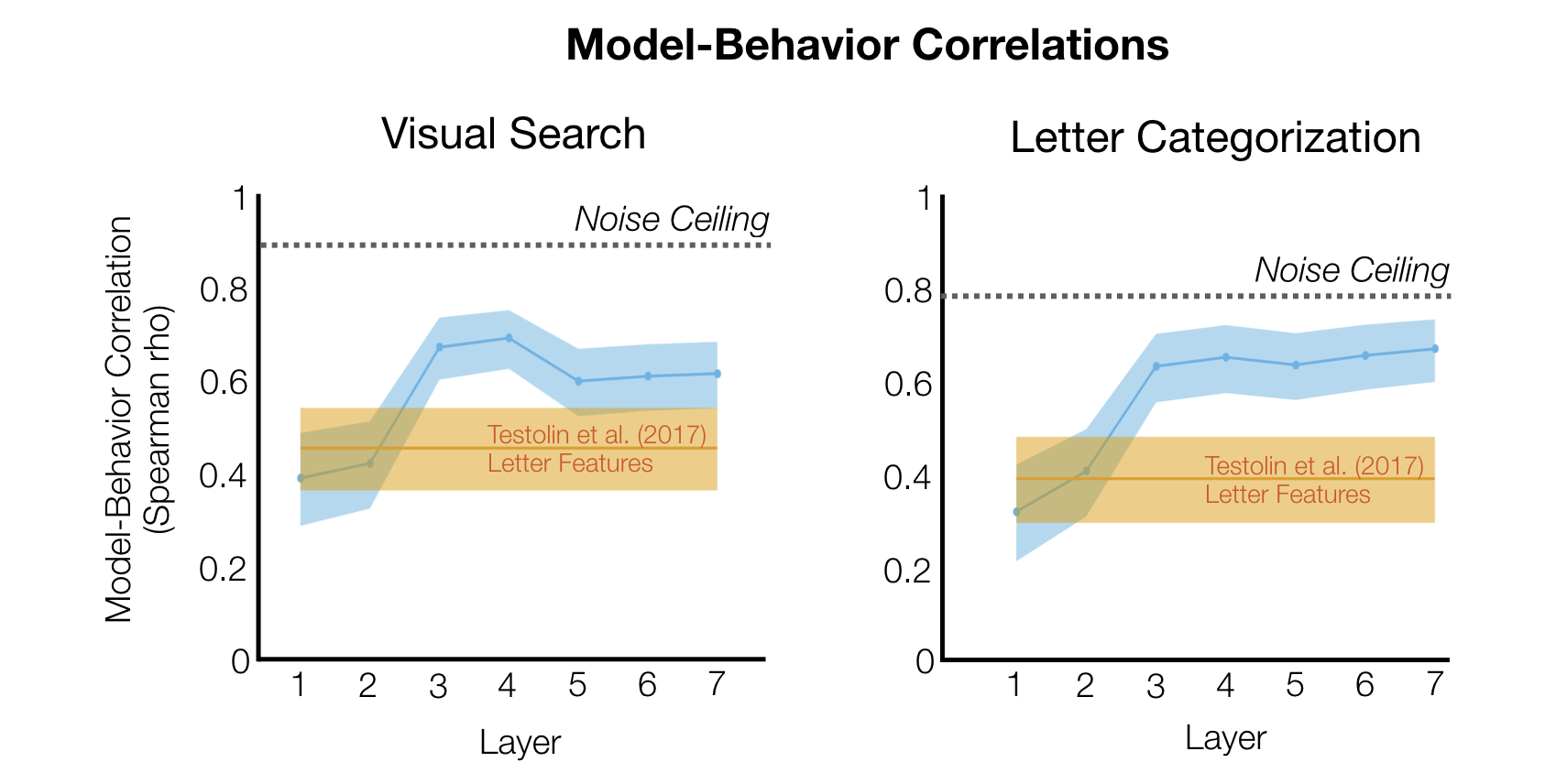

Supplement: S3 Fig — Model-behavior correlations are plotted on the y-axis, as a function of the layer of AlexNet trained on ImageNet. Model-behavior correlations for the letter-trained features from Testolin et al. (2017) are plotted in orange. The shaded error range indicate the 95% confidence interval across bootstrapped samples of letter pairs. (TIFF) [file pcbi.1010522.s004.tiff]

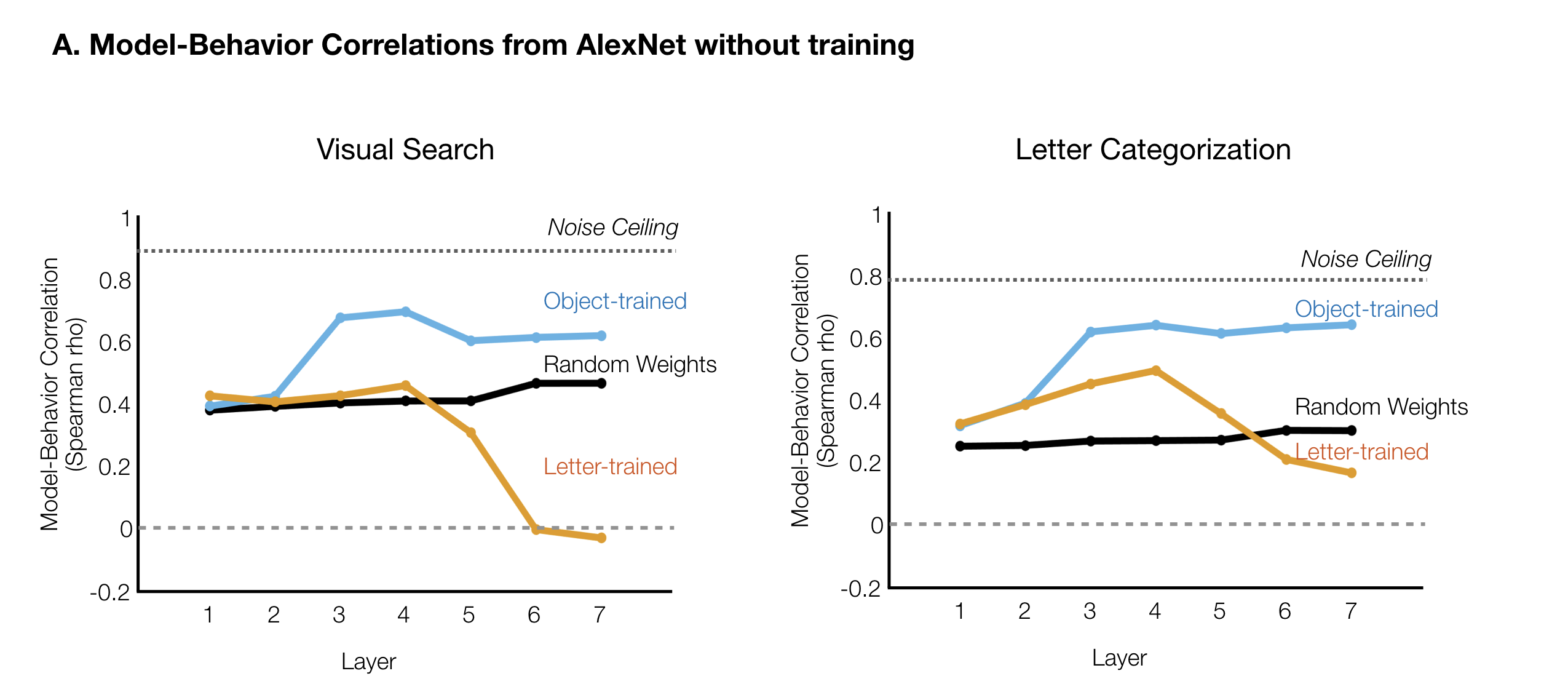

Supplement: S4 Fig — (TIFF) [file pcbi.1010522.s005.tiff]

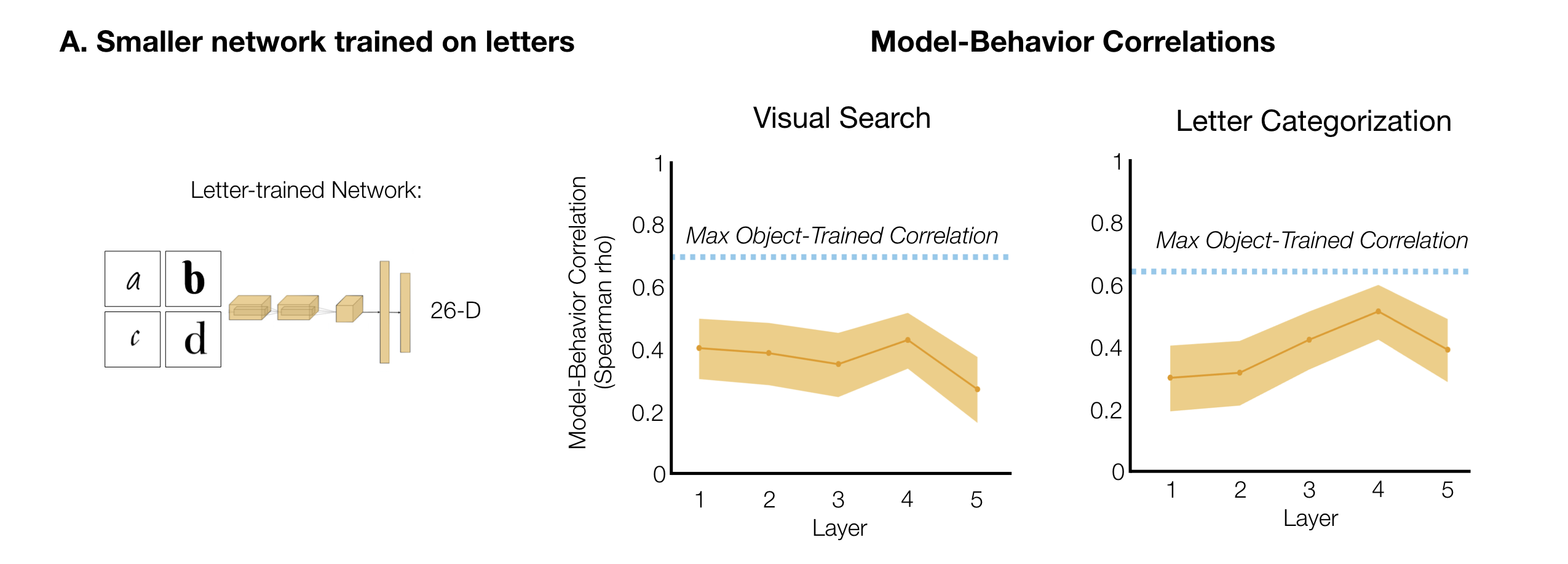

Supplement: S5 Fig — A smaller architecture (see Methods) was trained on 26-way letter classification to create another model of specialized letter features. Model-behavior correlations are plotted on the y-axis, as a function of the model layer. The shaded error range indicate the 95% confidence interval across bootstrapped samples of letter pairs. (TIFF) [file pcbi.1010522.s006.tiff]

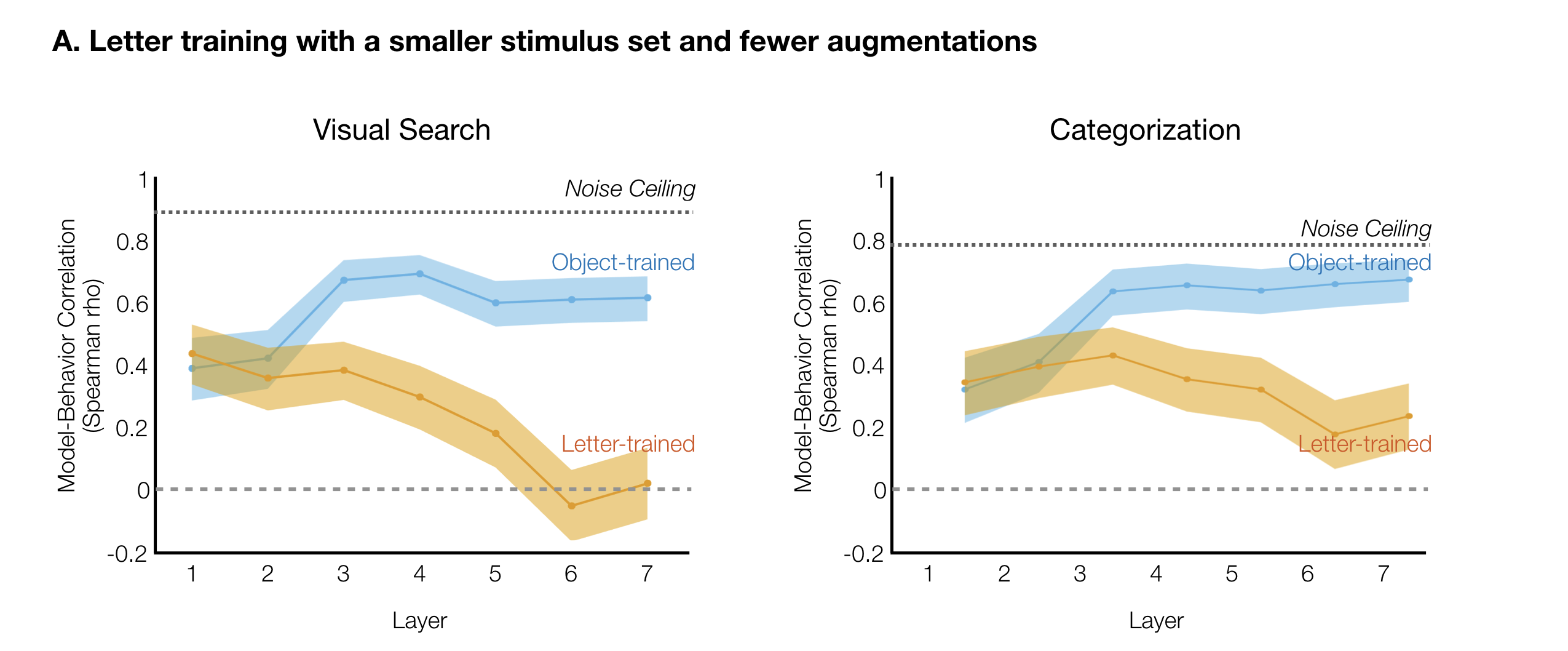

Supplement: S6 Fig — The letter-trained model shown here was trained on an image set with 550 typeset fonts per letter with size augmentation. In comparison, the letter-trained models in the main text were trained with 3344 typeset and handwritten letters across augmentations of size, position, skew, tilt, color, and noise. (TIFF) [file pcbi.1010522.s007.tiff]

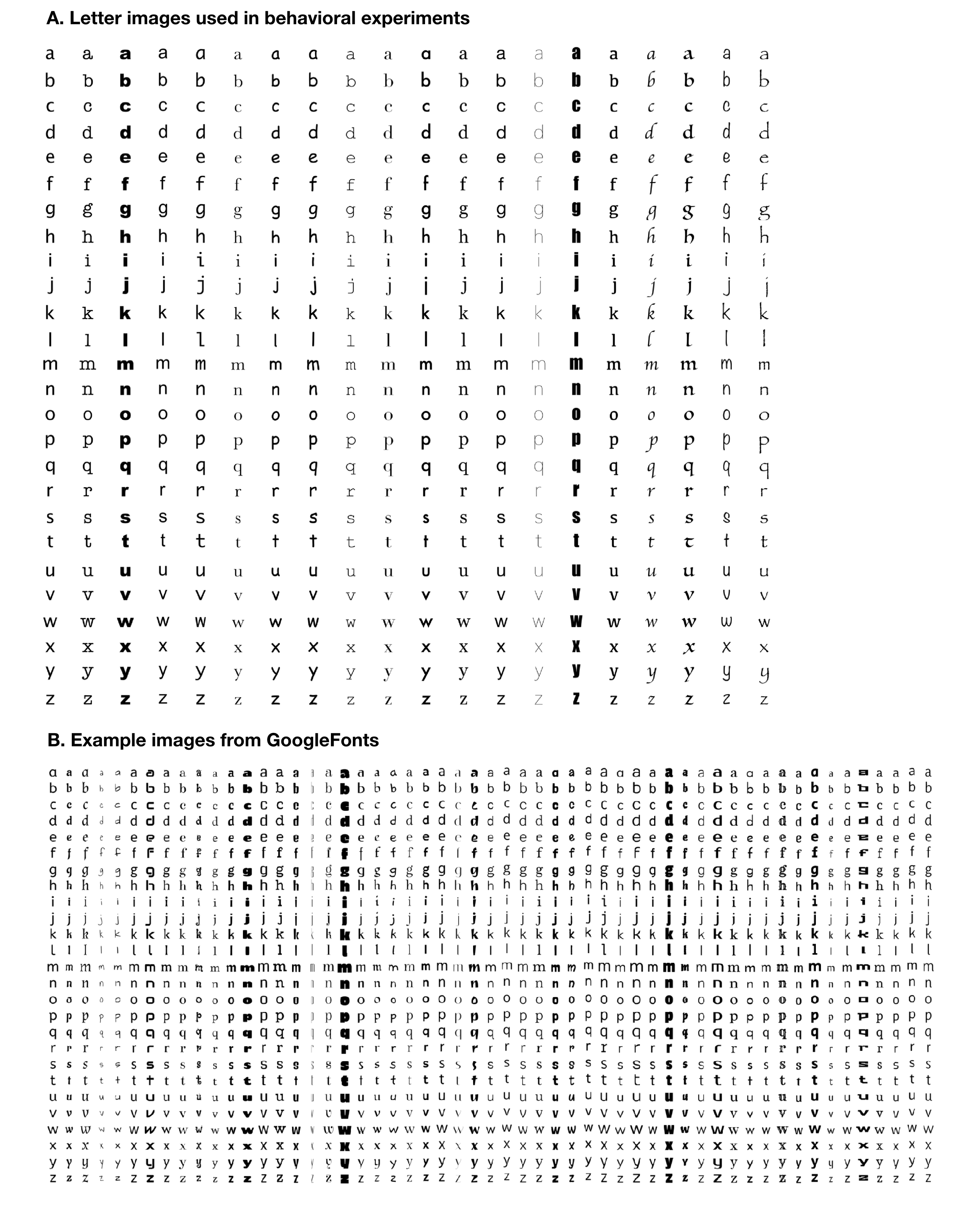

Supplement: S7 Fig — A. Images used in the two behavioral experiments: all twenty-six lower case letters across twenty fonts. B. Example images from the GoogleFonts database used to train specialized letter networks. The full database includes all twenty-six lowercase letters across 2344 fonts. (TIFF) [file pcbi.1010522.s008.tiff]

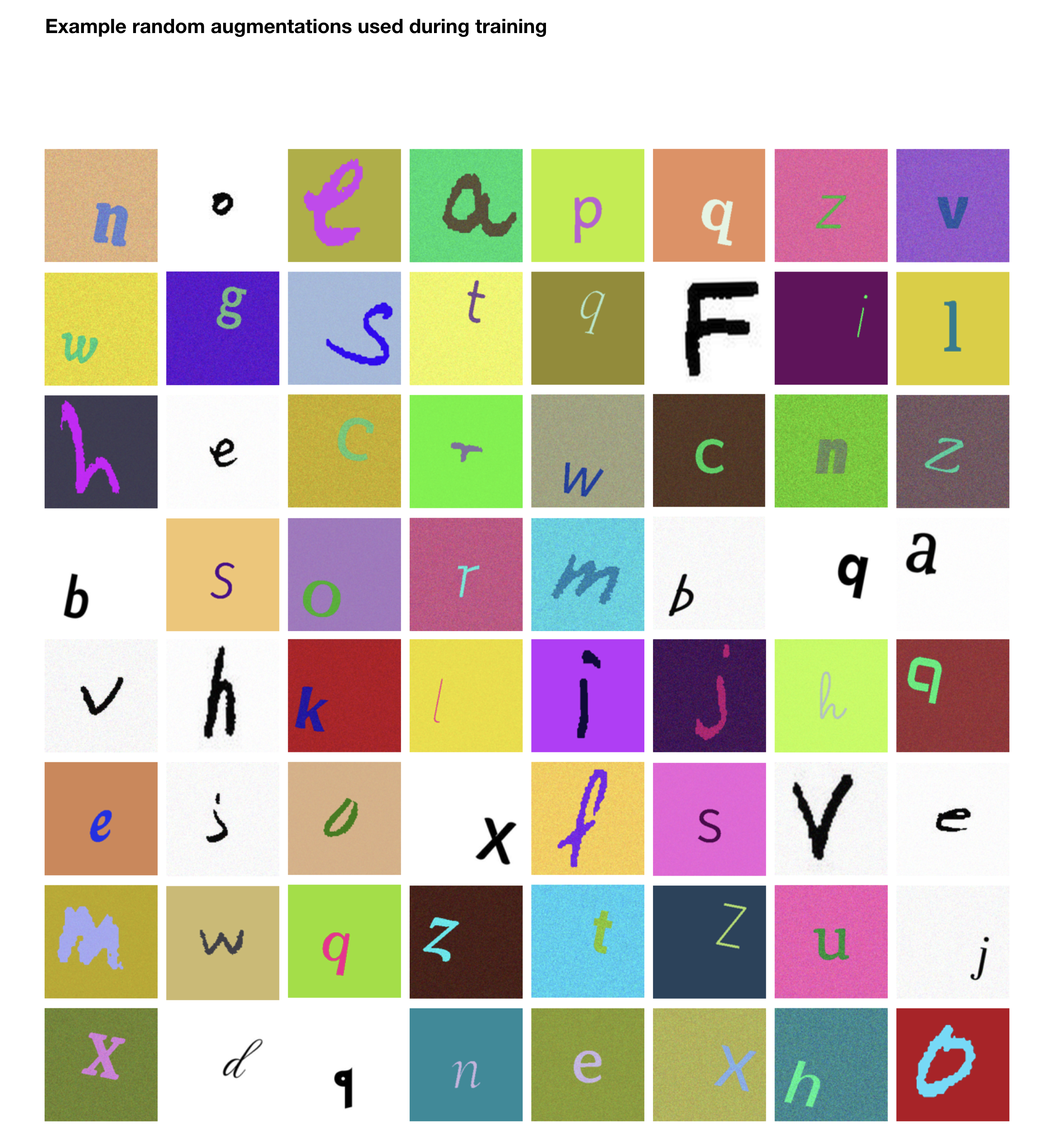

Supplement: S8 Fig — Augmentations included size, position, tilt, shearing, Gaussian noise, and color. For a full description of augmentations see the Methods section. (TIFF) [file pcbi.1010522.s009.tiff]
