# Supplementary material for: General object-based features account for letter perception
Source: PLoS Comput Biol. 2022 Sep 26;18(9):e1010522. doi: 10.1371/journal.pcbi.1010522 (PMC9536565; doi:10.1371/journal.pcbi.1010522)
Supplement: Attachment — Submitted filename: Janini_ResponseToReviewers_PLOS.pdf [file pcbi.1010522.s011.pdf]

HARVARD UNIVERSITY
DEPARTMENT OF PSYCHOLOGY

WILLIAM JAMES HALL
33 KIRKLAND STREET
CAMBRIDGE, MA 02138

Daniel Janini
daniel_janini@g.harvard.edu

Dear Editors,

We would like to submit our revised manuscript, “General object-based features account for letter perception”.

As you noted previously, the reviewers raised concerns about the format of our behavioral tasks, and the training of the letter network and its comparison to the object-trained network. In response to the reviewers’ comments, we clarified our interpretations of our results in relation to the structure of our behavioral tasks. In addition, we re-trained all letter classifying models with increased data augmentation and an expanded image set, and to add more context to our results, we added more model comparisons including pixelwise and untrained models. To include these additional analyses in the updated manuscript, we rearranged the order of presentation of some of the results (e.g., combining the results of the visual search and categorization experiments into one figure).

Most notably, the letter-trained AlexNet in the new manuscript exhibits higher correspondence to our behavioral data than in the previous manuscript. While ImageNet-trained AlexNet is still the best model at accounting for the visual similarity of letters, we have edited our wording throughout the manuscript from the title through the discussion section to encourage a more careful interpretation of the results.

We think these revisions have strengthened the paper in terms of robustness and clarity. We look forward to hearing from you about whether this work is acceptable for publication. A point-by-point response to reviewer comments is below.

Please don’t hesitate to contact us if you have any further questions. Thank you for your consideration.

Sincerely,
Daniel Janini, Chris Hamblin, Arturo Deza, and Talia Konkle

REVIEWER #1

In this paper, “General object-based features account for letter perception better than specialized letter features”, the authors tackle a timely and interesting topic in an interdisciplinary fashion. They perform large-scale behavioural experiments on letter perception and subsequently use normative modelling (i.e. task-trained convolutional neural network models) to better understand the patterns observed. They conclude that letter perception remains best explained by category-trained DNN feature spaces, providing an interesting perspective on neuronal recycling, and visual plasticity during reading acquisition. The paper is very clearly structured and was a pleasure to read. The figures are highly informative, and the available code will aid reproducibility. In short, I very much enjoyed this paper. Yet, I have a few concerns which I hope the authors will address in the resubmission to solidify the main claims. I very much hope that my comments will be perceived as constructive as they are intended and that they may help the authors improve their manuscript going forward. I look forward to our discussion.

Signed
Tim Kietzmann

- **Thanks for the positive feedback. We are glad that you enjoyed our paper!**

Major comments:

1. Both behavioural tasks, including the categorization task, seem rather perceptual in nature. This view is backed up by the high inter-experiment correlation reported in lines 290ff. As a result, similar letters are “close to each other” in the behavioural data. In contrast to this, the letter-trained DNNs are optimised for letter discrimination. As a result, perceptually similar letters will be projected onto feature axes that ideally distinguish between them. Given this observation, I am unsure in how far we should expect letter training to successfully capture data that relies on perceptual comparisons.

- **We agree with you that both behavioral tasks are perceptual in nature. We have added new text in the manuscript to clarify our thinking. When introducing the letter categorization task in the Results section, we have clarified that this a perceptual task (Lines 210-219). Additionally, we have added a paragraph to the discussion to explain the idea that both tasks involve operations over a common perceptual feature space (Lines 596-609). Further we have changed our use of the phrase “visual similarity” to “perceptual similarity” throughout the manuscript to make it clear that we believe both tasks measured a perceptual representational space.**
- **We consider both the object-trained and the letter-trained networks to be perceptual models because they each perform encapsulated transformations of sensory input. So, we believe in principle either model could have accounted for the perceptual tasks. The key question is how well different kinds of perceptual features correspond to the visual perception of letters – features ideally tuned to discriminate between letters, and more general-purpose features tuned to discriminate between many object categories. We have made edits throughout the manuscript to explain why we think it was plausible to consider the letter-trained network as a model for human letter perception. For example, see Lines 79-81 in the Intro, and Lines 213-216 in the results.**

- **To further affirm the plausibility that letter-trained networks operationalize a theoretically interesting hypothesis, we ask the reviewer to consider a hypothetical project which follows the same approach but for face perception. One could measure the visual similarity of faces through the same experimental methods found in this paper – measuring reaction time as participants distinguish between faces in visual search or categorize faces as quickly as possible. An experimenter could compare how well object-trained versus face-trained networks account for the visual similarity of faces. If participants distinguish between faces using domain-specific features rather than object features, then a model specifically trained to distinguish between faces may account for the behavioral data well. Indeed, neural networks trained on faces are often leveraged to investigate perceptual face representations (e.g. Aburdarham, Grosbard, and Yovel, 2021; Blauch, Behrmann, and Plaut, 2021; Yildirim et al., 2020). We believe that the same logic applies to this paper and letter specialization.**

2. The behavioural tasks and DNN analyses operate on rather different levels. Whereas the human data is collected via RTs, the networks internal representations are probed without a behavioural focus. Would it make sense for the authors to consider behaviour for both, models and brains? One way of doing this would be to train linear readouts on the various models in the tasks that humans engaged in, and to take the "distance to hyperplane" as a proxy for RTs.

- **We have added a paragraph to the general discussion to clarify how we believe the behaviorally measured RDMs relate to the representational structure measured in our neural network models (see lines 596-609). We consider visual feature spaces as placing encoding level constraints on behavior, without directly modelling the cognitive operations fully carrying out the behavioral task. We think this is a justified stance because the RDMs measured in the two tasks exhibit a high degree of similarity even though the two tasks are quite different in their cognitive process level requirements. This theoretical stance has been articulated in studies by Michael Cohen and we now reference this work in the Discussion as well.**

- **In addition, we added a section to the supplementary information to further explain some of the assumptions underlying the way we relate our neural network models to our behavioral measures. We have drawn attention to this information in the Methods section of the manuscript.**

- **In the context of our behavioral experiments, we did not necessarily think that distance to a hyperplane from a linear classifier would be an appropriate model of the read-out mechanism. For example, in the visual search experiment, participants had to identify the odd-letter-out without knowing ahead of time which two letter identities would be the target and distractor. Without knowing the letter identities being discriminated ahead of time, a participant could not apply a linear classifier for the two identities appearing in each trial.**

3. I am unsure how much we can trust the results of training AlexNet on comparably few letter data. AlexNet has ~61M parameters, but letter training was performed using only 14k datapoints. As a result, the model features will heavily overfit to the training data, likely amplifying my concern #1 above. In contrast to this, ILSVRC trained models have seen 1.3M images (~100x more data, not even considering the fact that the convolutional kernels benefit largely from input to all units in natural stimuli vs very sparse input for letters). Given this stark difference in the number of training samples, it is perhaps less surprising that model fine-tuning with 1026 instead of 1000 image categories led to comparably small changes in the model RDMs. In short, I feel like the models behave as they should. ILSVRC training will be quite similar to ILSVRC+letters, and training on letters only will lead to a model that can easily distinguish between them, thereby leading to a worse fit to tasks that rely on perceptual similarity.

- **You raise the concern that our networks trained on letter images alone have a small amount of data relative to number of network parameters being fit. Indeed, we also trained a smaller architecture on letter classification for precisely this reason, where there are fewer concerns of over fitting, because of the smaller number of network parameters. We have added a sentence to the results section to clarify this point. However, to more pro-actively consider this concern, in the new manuscript we trained new models with more images and varied data augmentation. We found that these decisions mattered and improved how well the letter-trained model corresponded to the behavioral data.**
- **More specifically, we returned to the Google Fonts web service and found that more fonts were available than before, so we added these to the image set. In addition, we included variations of bold and italic fonts. We also included images of handwritten letters from the NIST dataset. Altogether this increased the number of images per letter category from 550 to 3340. We also included a wider variety of augmentations during training (size, tilt, position, noise, shearing, and color). We have updated our methods section to reflect these changes. We retrained our letter models, finetuned models, and branching models with these extra data and augmentations, and updated all results and figures based on these new models.**
- **The expanded image set and augmentations did change the representational spaces of the letter trained network. In comparison to the previous draft, the middle layers of the letter-trained network now exhibit higher correlations with the visual search and categorization data. In both experiments, the object-trained network still exhibited the highest correspondence to the behavioral data, and showed more consistent correspondences to the behavioral data, but the size of this effect is diminished. As such we have changed our wording throughout the manuscript to encourage a more careful reading of the results.**
- **In addition, we now note in the Results section the importance of considering augmentations when training models.**

- **You stated that are not surprised that model finetuning on 1026 categories instead of 1000 categories did not drastically alter the model RDMs, because there were such fewer letter images than object images. In the revised manuscript, we now clarify that while the finetuned model did exhibit highly similar RDMs to the ILSVRC trained model in Layers 1-5 (range r=0.795-0.995), the RDMs from the fully connected layers exhibited less similarity between the two models (r = 0.68 and r = 0.57 in Layers 6 and**

7, respectively). This made it possible for the finetuned model to improve upon the ILSVRC trained model in its correspondence to human behavior, but instead we found that the finetuning did not help. This information has been added to the results under the section "Models of object-based features with experience dependent specialization".

Minor Comments:

1. Very small detail, but it could be clarified that AlexNet was trained on ILSVRC 2012, which represents only a small fraction of ImageNet.

- **We have added a sentence to the methods section to clarify this point: "More specifically, AlexNet was trained on the subset of ImageNet used in the 2012 ImageNet Large Scale Visual Recognition Challenge (78)."**

2. Could I ask the authors to clarify how exactly the model RDMs were computed? The methods section states that "we computed the summed activation map of each feature for each stimulus", but it remains unclear to me what actually happened. The more traditional approach would be to take each network unit as its own dimension, but this seems to not have happened here. Moreover, could the authors comment on whether RSA-reweighting (e.g. Storrs et al.) would make a difference to the results obtained?

- **We have clarified how we computed model RDMs in the methods section (Lines 870-872). We have also added justification for this approach in Lines 872-878.**

- **We did not conduct RSA-reweighting in this study, primarily because we think that it is a higher bar for feature spaces from neural network models to exhibit high model-behavior correlations without reweighting. We added this reasoning in the methods section (see Lines 919-923).**

REVIEWER 2

In manuscript reports a study investigating hypotheses about whether letter representation depends on learning to recognize special letter features or by reusing general visual features acquired through object recognition. Similarity judgments for letters (visual search and visual categorization) were compared to the layer representations of neural networks either trained to do standard 1000-class image classification or 26-class image classification. Using representational similarity analysis, it was found that the later layers of the object-trained networks showed better correspondence to the behavioral data for both tasks. The authors interpret these results in favor of the domain-general learning and reuse hypothesis.

I think this is a topic in which NNs do provide a useful tool for evaluating different hypotheses. Overall, I think it is a great project to be pursuing. I have some serious concerns though about how the results are interpreted and whether enough has been done to really show that an object-trained network is superior to one specialized for representing letters. As outlined in some of my major comments, I think a lot more work would need to go into how the letter

network is trained in order to make a reasonable comparison to the object trained network. Along these lines, below are several comments/suggestions about analyses that could be carried out (some simple, some rather laborious). I hope the authors find them to be constructive. Ultimately, I think the conclusions that can be drawn from this study are, at present, rather limited as are the findings.

- **We thank you for your comments. We are happy to hear that you think neural networks provide a useful tool for the hypotheses in this study, and that this is a good project to pursue. Following your comments, we have put forth substantial effort in training the letter network, and we have edited the manuscript to clarify the scope of our findings. A detailed response follows.**

A. The authors say (L132-133):

   “These results provide clear empirical support for the theoretical position that letter perception is primarily supported by general object-based features, rather than specialized features.”

   I do not see how these sorts of results could do this, even in principle. Taken at face value, the results show that the object recognition trained network better captures the letter similarity judgments and so it is *possible* to have an architecture that might represent the similarity of letters without being trained to represent a letter feature space. Showing this possibility is an interesting, if modest, result. But showing such a possibility is not sufficient as evidence in favor of the psychological hypothesis that letter perception is “actually” generated by such a learning process. Especially not when an ImageNet trained network does not even obviously “see” global shape (see comment below), which is presumably required for representing letters under either of the hypotheses considered.

   To be clear, I am extremely sympathetic to the reuse hypothesis that the authors think their results support. I just don’t think it gets support from these results. At most this is a kind of proof of principle for an aspect of the hypothesis.

- **You’re correct that this study cannot fully arbitrate on the hypothesis that general object features are reused to support letter representations. We have attempted to clarify our language in the manuscript to this end. For example, we have changed the wording of the quoted passage to soften the claim made. Instead of saying we provide “clear empirical support”, we now state that our findings “lend computational plausibility to the theoretical position that letter perception is supported by general object-based features”. Throughout the manuscript we have scrutinized the phrasing to clarify the kind of evidence that we are providing without implying that the question has been conclusively settled.**
- **We do believe that by comparing computational models to empirical data, our work supports the plausibility of a particular theoretical position (that letters could be perceived via features that are not directly about letters per se). Prior to the advent of neural networks capable of performing well on a variety of classification tasks, researchers could not computationally assess the plausibility of this domain-general hypothesis, so we believe our study provides a new aspect of plausibility for this theoretical position.**

B. There are a few analyses that I think would be useful for clarifying these results, which would not require much additional work by the authors.

1. First, how do the results look if one looks font-by-font, across the 20 different fonts? That is, makes a different 26 x 26 matrix for each of the 20 font, rather than averaging (L752-L753). This would be useful for showing the robustness of the results; that the performance is consistently higher across fonts.

- **We conducted the analysis suggested here, and we found that the object-trained network exhibited higher model-behavior correlations than the letter-trained network at the font-level as well. This result has been added to the revised manuscript in Lines 195-198. We agree with the reviewer that this supports the robustness of our results.**

2. Second, I think it would be useful to have a baseline model of the stimulus properties to compare to the behavioral judgments and neural data. For example, make 26 x 26 pixel similarity models of each font, and then average all 20 of these matrices to get the total pixel similarity model. How much would the observers performance be predicted by this model on the two tasks? Does it better capture the representation of the two NNs? This might also help clarify how they are each representing things.

- **We have conducted this analysis and included it in the results section. We found that the visual similarity of letters is only moderately accounted for by the pixelwise similarity of letters. The correlation between the visual similarity of letters and the pixelwise RDM was $\rho = 0.39$ for Experiment 1 and $\rho = 0.43$ for Experiment 2. We have also visualized these results in Figures 1B and 1C.**
- **In comparison to the pixel model, both the object-trained network and letter-trained network learned feature spaces which better corresponded to the visual similarity of letters. We now report these results in the revised results section as well.**
- **Comparisons between the two neural networks and the pixelwise model did not reveal major differences. RDMs from both neural networks exhibited decent correlations to the pixel RDM throughout all layers (ranges: $r = 0.48$-0.67 for the letter-trained model, and $r = 0.48$-0.59 for the object-trained model). Correlations to the pixel model between the two networks did not show marked differences based on layer depth. Thus, at every representational stage of both models, representational structure from the pixel input was maintained to some degree.**

3. Third, I think it would be helpful to have a baseline NN as well. For this, maybe see what results one gets with a completely untrained model, since even untrained DNNs have shown some correspondence to neural responses. Might help the authors case if an untrained model even does better than the letter trained model!

We have included this analysis in the revised results section and we visualize the results in Supplementary Figure 4. We found that in comparison to an untrained model, both the object-trained model and letter-trained model had higher correlations with the visual similarity of letters in both Exp1 and Exp2.

C. Beyond the issue raised in A, comparing the networks in this study really seems like apples and oranges. The nature of the two sets of training images are so radically different in their properties, in ways that we know matter to the training and generalization of NNs, that there could be many reasons for explaining the difference in performance that has little do with the hypotheses under consideration. Unless I missed it (and please, correct me), the only variation used was in the fonts used (550 total). But what about:

- **We thank the reviewer for considering a wide variety of ways in which our models could be trained to improve the generalizability of their representations. Before we respond to each suggestion one at a time, here we explain some broader considerations underlying our choices.**
- **First, we believe that the most important requirement in this study when comparing a letter-trained and an object-trained model is that they both have feature spaces which can support letter classification in a linear way. We found that this was the case for both of our models. That is, each of these models represent and distinguish between letters accurately, both in a test set of letters and in the experimental stimuli themselves. However, the two models distinguish between letters in different ways, thus operationalizing different representational hypotheses. We have clarified this point in the results (see Lines 161-181)**
- **Second, to operationalize letter-specific features we believe it is important that a letter trained model is not trained on images including objects or their features. This would create the possibility that the model learns the features of objects to classify letters.On this point, we have added clarification to the Introduction (see Lines 118-120) and Methods section (see Lines 783-785).**

-Variation in the size, position, contrast, rotation in the picture plane, or even rotation in the depth plane. Addition of noise (cf. Testolin et al. 2017).

- **In our original models, the letter-trained models in our previous draft did include size variation in addition to font variation. In the revised manuscript, we have trained new letter models with expanded augmentations including variations of size, position, tilt, shearing, noise, and color. We did not include rotation in the depth plane.**
- **In addition, we increased the number of letter images used to train our models. We added more fonts from the Google Fonts we service, and we also included images of handwritten letters from the NIST dataset. Altogether this increased the number of images per letter category from 550 to ~3340.**
- **The expanded image set and augmentations did change the representational spaces of the letter trained network. In comparison to the previous draft, the middle layers of the letter-trained network now exhibit higher correlations with the visual search and categorization data. The object-trained network still exhibited more consistent correspondences with the behavioral data, but the size of this effect is diminished. As such we have changed our wording throughout the manuscript to encourage a more careful reading of the results.**

-The presentation of the characters in natural scene backgrounds in which they have been convolved but are still discriminable.

- **We chose not to train the letter network in this way because of concerns that it would learn scene features. We believe such a model could hypothetically learn letter identities by learning interactions between scene features (e.g., gist features) and letter shapes, diminishing how well the network operationalizes letter-specific features.**

-What about training them to do classification of 26 letters x 20 behaviorally tested fonts. This would be more similar to what you have in ImageNet with different breeds of dogs, rather than all dogs being treated as alike. The 520 different categories would then at least give some semblance on similarity in scale of the classification task being performed.

- **We did not train our model in this way, because we believe it is important that the model learns to distinguish between letters in a way that mirrors our experimental tasks. In the visual search task, participants distinguished between letter identities, and in the categorization task they distinguished between identities across trial-by-trial variation in font. Thus, they did not distinguish between the same letter in two different fonts (e.g., "a" in Helvetica versus "a" in Comic Sans). We wanted our letter model to similarly learn features tuned for classifying letters by their identity.**
- **In addition, training a model in this way would amount to training on a small sample of fonts, and each category would be learned from a single image across a variety of augmentations. We instead thought it was best to opt for a wide variety of images per category (~3340) with a wide variety of augmentations.**

-To really test this, you would need an ImageNet *of letters* in which letters appear in natural scenes with natural variation in viewpoint, illumination, partial occlusion, and so on and so forth. One might even just take pictures of pages of the sorts of books that we use to teach children letters. But I think there would have be some sort of matching of natural variation in the images to make an object trained network and a letter trained network comparable.

- **It is an interesting idea to train a model over pictures from natural environments like picture books. In the revised manuscript, we believe the additional images and augmentations provide many of the same variations in input that children typically learn letters from.**
- **The augmentations of tilt, size, shearing, position, color, and noise variation mirror certain natural variations in viewpoint and illumination.**
- **The addition of handwritten letters to the data set also provides another form of letter input that children typically learn from.**

-One suggestion in line with the previous comment: do "stylized" letters like Geirhos et al. (2021) do for objects to improve representation of shape.

- **If we used style transfer to add the style of depicted scenes with objects to letters, then the image set would contain features from both objects and scenes. Thus, this would also operationalize a feature space that is more of a hybrid of letter-object feature space. We chose not to go down this route because it is not clear what feature-based hypothesis this kind of model would operationalize (see also response below).**

D. The authors cite studies that highlight the difference between how humans and DNNs represent objects (citations 67-71). Some of those studies though point to the ways in which DNNs do not really represent global shape (e.g. Baker et al. 2020, Geirhos et al. 2021). In order for the comparison of models to be most informative, I think one cannot be just relying on standard ImageNet trained networks unless it is just to make the “mere possibility” claim, per comment A above. I think one would have to do something to try and show that the networks are actually representing shape properties, since under anyone’s view, letters are represented as complex two-dimensional shapes on surfaces (or so I would hope).

For example, to add a suggestion to the list from comment C above, suppose one made a stylized-Letters image set a la Geirhos et al. 2021. Their results suggest that a network trained on stylized object images better represents the actual shape. In fact this is someone one could do for both the objects and letters, stylizing in similar ways. This would make the image sets more comparable, and if the object trained network was still better, then this would indeed support the possibility claim from comment A above.

- **Following your suggestions, we explored the representational features learned in Geirhos et al.’s stylized-ImageNet-trained AlexNet, and we added the findings in the results section. Stylized-ImageNet-trained AlexNet did show slightly higher correlations with the visual search data ($p = 0.034$), but it did not differ from typical-ImageNet-trained AlexNet for the categorization experiment ($p = .158$). These results suggest that a model with more global shape features also corresponds well with the visual similarity of letters, though we didn’t find evidence that it was consistently better than a model with predominately local shape features.**
- **As stated in our response to part C, we did not train a model on letter categorization with stylized letters. Such an image set would contain features from both objects and scenes, so it would not operationalize letter-specific features.**

- **Your comments on shape representations prompted us to clarify our reasoning in the manuscript. We have now added material in the Discussion section to spell out what we know about the nature of the features in object-trained neural networks, and why we think these are appropriate (though imperfect) candidate models of human object representations (see Lines 576-588).**

- **We agree that letters are represented by shape features. However, shape can be operationalized in many ways. For example, it is uncertain the extent to which objects and letters are represented by local versus global shape features. Rather than viewing letter representations as being dependent on global shape, experimenters studying the perception of letters often assume letters are represented by a host of local contour features (e.g., line orientations, terminations, and curves; see Fiset et al., 2008; Wiley, Wilson, and Rapp, 2016).**

- **Moreover, neural networks trained on object categorization do represent shape properties, even if these shape properties are predominately local contour features rather than holistic/global shape features (Baker, Lu, Erlikhman, Kellman, 2020). We believe it is informative to leverage the hierarchical feature spaces learned in DNNs, even if they lack global shape processing. Indeed, some recent research has argued**

that high-level visual areas are more sensitive to local shape and texture information than the global form of objects, mirroring the representations of DNNs trained on object recognition (Jagadeesh and Gardner, 2022). Thus, we believe there is active debate about the extent to which objects and letters are represented by local versus global shape features. Insofar as objects and letters are represented by local shape features in the human visual system, we believe that CNNs are suitable models.

Minor Comments:

Capitalization for figure panels changes (L152 vs L156).

- **We have fixed this capitalization.**

L186 "First, visual feature spaces" ?

- **We have made this change for clarity.**

If the similarity is going to be plotted for visual search (Figure 1B) it should be plotted for the categorization data in Figure 2.

- **In the updated manuscript we include results from the visual search and categorization experiments in Figure 1. We have now included the MDS plot for the categorization experiment in this figure as well.**

-What are the between task reliabilities when split by font? I think it would be helpful to report this in the supplemental.

- **In Experiment 2, each participant could only complete a small sample of all the possible letter pairs, so we used linear mixed effects modelling to estimate the reaction time for each pair of letters across participants. Splitting each participant's data by font would not allow for reliable estimates of the 26x26 RDM in each font. Unfortunately, this prevents us from comparing the 26x26 letter RDMs between our two tasks.**

-I wonder if some of the visualization in Sup. Figure 3 could/should be in the main text.

- **We decided to continue to include these model MDS plots in the supplementary text because not all high dimensional model feature spaces were able to be faithfully reduced to two-dimensional displays. We believe the figures still allow for exploratory inspection, but we did not want to mislead readers by elevating the figures into the main text.**

-I might have missed it, but what were the layer-by-layer correlations between the object and letter networks?

- **This information has been added to the main text and Supplementary Figure 2. The two models showed the following correlations between their RDMs:**

Layer 1	Layer 2	Layer 3	Layer 4	Layer 5	Layer 6	Layer 7
0.98	0.86	0.76	0.83	0.61	0.41	0.37

-L473 Although it is tantalizing to know that there is an ongoing fMRI study on this, I think this sentence can be cut.

- **We have removed this sentence in accordance with this feedback.**

Have the authors made all data and (if applicable) computational code underlying the findings in their manuscript fully available?
Reviewer #2: **No:** Only the behavioral data and stimuli are up on OSF. They could have at least uploaded the network RDMs.

- **We would like to clarify that all necessary data and code for reproducing our analyses are included on the OSF repo, including model activations and RDMs. The other reviewers indicated that they were able to access the data and code, so let us know if you continue to have a problem accessing them!**

REVIEWER 3

Janini and colleagues investigate the representational space of letter perception in humans using behavior and computational approaches. They find that a neural network trained on general object discrimination matches human behavioral performance on two letter discrimination tasks better than a neural network trained to discriminate letters specifically. The results provide strong support for a domain-general account to letter perception. These data provide important insights into how humans learn and perceive letters. This study will be of broad interest to the cognitive psychology and neuroscience community. Overall, this is a very nice study. Aspects of the analytic approach and data need clarification and further illustration. The authors should better address the degree of task dependency of their results. The discussion section would benefit from discussion of case studies and clarification on the broader impacts of the results.

- **Thank you for your kind comments!**

Specific comments

1. The finding of correlations between behavior and the object-trained model close to the noise ceiling provides good support for the authors' domain-general account of cortical recycling in letter perception. In general, I agree with this interpretation, but the task demands are particularly important here. Would a task that is geared more towards word reading support

a similar conclusion? This is particularly relevant when discussing the current results in relation to the VWFA. Also see the next comment re: behavior of individuals with alexia.

- **We thank the reviewer for bringing up this insightful question. We have edited the discussion to consider this question in the following paragraph:**

"In this study, we chose to focus on individually presented lowercase letters in the Roman alphabet, raising the question of whether our claims would differ if our behavioral tasks and models were aimed at letter-string and word-level representations. This would be the case if the visual system learns specialized features for detecting combinations of letters en route to whole word representations (Vinckier, 2007). However, focusing on individual letter-representations is not wholly unjustified, as there is some empirical support that letter string representations are simply linear combinations of letter representations (Agrawal et al., 2019). For example, the visual similarity of bigrams as measured through visual search is linearly predictable from the visual similarity of their constituent letters (Agrawal et al., 2019). In addition, our work does not address the perception of non-alphabetical writing systems. Future behavioral-modelling work could leverage the approaches used here to investigate the extent to which other written symbol representations rely on general versus specialized feature spaces."

2. Overall, there is good discussion of how the results relate and add to prior literature. Additionally, I would like to see discussion of the relation to alexia arising from damage to the VWFA (e.g. Turkeltaub et al. 2014 Neurocase, also Behrmann et al. 1998 Neuropsychologia) that can result in letter-by-letter reading. These lesion cases are highly relevant for the discussion on domain-general vs -specific and (lack of) localization to a "letter subspace".

- **We thank the reviewer for asking us to consider neuropsychological research relating to the domain-specificity versus -generality of orthographic representations. We found this comment intertwined with the issues raised in Comment 8, in which the reviewer felt that we appealed to two conflicting perspectives on domain specialization, dampening the impact of our study. We have edited the discussion to include the following paragraph which we believe clarifies these different perspectives on domain specialization:**

"While our work here makes inferences about the nature of visual system representations through a behavioral-computational approach, our findings also lead to predictions about the representations of the visual word form area in the ventral stream. Neuropsychological studies have found that damage to the left occipitotemporal cortex (in the vicinity of the visual word form area) leads to slow and laborious letter-by-letter reading of words, though debates surround the extent to which such patients also show deficits in object perception (63,64). In addition, studies on the connectivity constraints between language regions and occipitotemporal cortex support the view that information on letter strings is preferentially read-out from the visual word form area (65–69). These neuropsychology and connectivity studies help arbitrate on whether causally relevant information on letter strings is represented in a local region of visual cortex. However, these findings do not directly speak to the nature of the feature tuning in this region - whether the features are specifically tuned to distinguish between letters, or if they are more generally tuned to distinguish among all visual inputs. Based on our behavioral

findings, we make the following brain predictions about responses to letters in the visual word form area. First, we predict general object-based features will account for the representational space for letters both before and after letter learning. Second, we predict letter learning will not create new patterns of response to letters in the visual word form area. Instead, we predict that letter learning will increases the amplitude of those patterns, facilitating read-out by increasing their signal-to-noise ratio."

3. The decrease in model-behavior correlations at higher layers for the letter-trained network is striking. Presumably this network does just fine at discriminating letters. Some investigation into what's going on would help in interpreting comparisons with the object-trained network. e.g. looking at the MDS plots in Supplementary Figure 3, letters appear to be more dissimilar from one another in the letter-trained network. I wonder if there is an interesting story here about capacity limits to cortical feature spaces.

- **You are correct that the letter-trained network does just fine at discriminating letters. Previously we brought attention to this in the methods section, but we have clarified this in the revised manuscript by mentioning this in the results section when first introducing the model (see Lines 166-167).**
- **We were not certain how to pursue the ideas about capacity limits of feature spaces directly with these data, but we have made all model RDMs and behavioral data available on the OSF repo, so a reader wanting to explore this idea could do so.**

4. How does fine-tune training affect object-trained networks? How different are the RDMs after training? What was the accuracy for letter discrimination in these networks compared to the letter-trained network?

- **In the revised manuscript, we have added an explanation of how finetuning affected the RDMs of AlexNet (see Lines 311-316). Our primary finetuned model (the 1026-way object and letter classifier) had highly similar RDMs to the pre-finetuned model in Layers 1-5 (range r=0.795-0.995), the RDMs from the fully connected layers exhibited less similarity between the two models (r = 0.68 and r = 0.57 in Layers 6 and 7, respectively).**

- **Because the object-trained network was not trained on letters, we assessed its ability to support letter classification by training linear classifiers over its feature spaces (as described in the Results section Lines 161-162 and in Supplementary Figure 1). Layers 3-7 of the object-trained network exhibited 95-97% accuracy at pairwise decoding of letter identities across font and size variation. The letter-trained network had a Top 1 accuracy of 98.5%, while the fine-tuned network had a Top 1 accuracy of 98.4%. These accuracies are listed in Supplementary Table 1.**

5. How (dis-)similar are letters across fonts (both for trained and tested)? I would expect changes in the representation space from learning to be strongest for letters that that have a good degree of variability across fonts. Given that much of the analyses focused between letter comparisons within a font or mean activations across fonts, would analyses targeted on variability across fonts reveal letter-specific effects of learning?

- **Unfortunately, our behavioral experiments were not designed to reliably measure differences between exemplars of the same letter identity across font (e.g., the similarity between “a” in Helvetica and “a” in Comic Sans). This limits our ability to directly address this question. We did come up with one analysis that indirectly explores this question though, which we describe below:**
- **We considered the possibility that letters with more pixelwise variation by font are better accounted for by specialized features. We first determined how much variation in pixel input each letter identity has across font (g, m, and p have the most variation and i, l, and v have the least). Then for each letter, we computed its model-behavior correlations with the object- and letter-trained networks. More specifically, we selected each letter’s values in the behaviorally measured RDMs (e.g., for “a”, a-versus-b, a-versus-c, etc), and then computed its correlation with the model RDMs. This provided a range of letter-specific model-behavior correlations for each layer of the letter-trained network. Finally, we calculated the correlation between the variation in pixel input of the letters and their model-behavior correlations. If the specialized letter model better captures representations of letters with a high degree of input variability due to font, then there should be a positive correlation between pixel variation and model-behavior correlation. Instead, we found low correlations across all layers that were not consistently positive or negative (range: r = -0.40-0.38 for Experiment 1, range: r = -0.36-0.35 for Experiment 2). Thus, from this analysis we did not find evidence that letters with higher variance in pixel input were particularly well accounted for by the letter-trained model. We have chosen not to include this exploratory analysis in the revised manuscript because we find it to be a fairly indirect test of the hypothesis.**

6. Relatedly, (as I understand it) the model RDMs for experiment 2 were computed by first calculating the mean activations for each letter across fonts. This doesn't appear well matched to the experimental design where subjects indicated whether single letters at specific fonts matched a target letter. Wouldn't it make more sense to calculate the distances between activations for font-specific individual letters compared to the target?

- **We agree-- it makes more sense to calculate the distance between activations for font-specific letters with the target letter. In the revised manuscript, we now use this method, and we have updated our methods section to reflect these changes.**

7. How do model-behavior correlations for the letter-preferring subspace compare with correlations for non-letter preferring subspaces (matched in # of units to letter-preferring)?

- **We have now conducted this analysis and included it in the results section. The maximum model-behavior correlations did not differ between the letter-preferring subspace and samples of non-letter-preferring features matched in # of units (*p* = 0.1824 for Exp1, *p* = 0.0812 for Exp2).**

8. Overall, the discussion section is well written, but the claim of a fully domain-general account of letter perception (p 21) appears at odds (and undercut) by the later discussion of possible domain specialization (page 24). This comes across as trying to appeal to two (often conflicting perspectives) on cortical specialization and dampens the impact of the study.

- **We hope that we adequately addressed this comment in our response to Comment 2 above. In short, we have now tried to clarify in the manuscript the difference between studies which arbitrate on whether letters are represented in a specific region of cortex versus studies like ours which help arbitrate on the nature of the feature tuning underlying our perception of letters.**

9. The authors propose that letter perception is supported by fully domain-general representations whose features are unaltered by letter learning. Why is this still considered cortical "recycling"?

- **Our understanding is that a variety of learning processes are compatible with the "neuronal recycling hypothesis" given how it has been articulated by Dehaene and colleagues. At the end of the second paragraph of the introduction, we state our understanding of this hypothesis: "a recycling process could leave the object-based features unaltered, or it could alter the object-based features with some degree of experience-dependent specialization." Under this understanding, learning linear read out mechanisms over general object features is one process in alignment of the neuronal recycling hypothesis.**

Minor comments

1. What was the behavioral accuracy for both experiments? Was accuracy and RT correlated across letter pairs?

- **In Exp1, the mean accuracy was 97.5% with a standard deviation of 1.7%. In Exp 2, the mean accuracy of included participants was 97.2% with a standard deviation of 2.0%. This information has been added to the results.**
- **In Experiment 1, only a moderately reliable RDM could be measured based on accuracy (ρ = 0.44, in comparison to a reliability of ρ = 0.89 for the RT-based RDM). The accuracy-based RDM was moderately correlated with the RT-based RDM (ρ = 0.47), in line with the lower reliability of the accuracy-based RDM. In Experiment 2, the accuracy-based RDM also had moderate reliability (ρ = 0.52). The accuracy-based RDM and RT-based RDM had a correlation of ρ = 0.55, again a comparable magnitude to the reliability of the accuracy-based RDM itself. We have added this information to the Methods section of the manuscript.**

2. For the neural networks trained on letters, what was the accuracy in discriminating letter pairs?

- **The accuracy of all letter-trained networks on letter classification can be found in Supplementary Table 1. In the revised manuscript we have called attention to this table in the Results and Methods sections.**

3. Please include the input layer in the model-behavior correlation plots.

- **We have updated our figures to include the input layer.**

4. I may have missed this in the methods - how were target letters given for experiment 2? Auditorily or visually? If visually, what font was used?

- **In the instructions, target letters were given via visual text in font Arial, along with the rest of the written instructions. Participants expected font variation in practice trials. We have updated the methods section to include this information.**
