# Supplementary material for: General object-based features account for letter perception
Source: PLoS Comput Biol. 2022 Sep 26;18(9):e1010522. doi: 10.1371/journal.pcbi.1010522 (PMC9536565; doi:10.1371/journal.pcbi.1010522)
Supplement: Attachment — Submitted filename: PLOSRevision-ResponseToReviewers.docx [file pcbi.1010522.s012.docx]

HARVARD UNIVERSITY

DEPARTMENT OF PSYCHOLOGY

WILLIAM JAMES HALL	Daniel Janini

33 KIRKLAND STREET	[daniel_janini@g.harvard.edu](mailto:daniel_janini@g.harvard.edu)

CAMBRIDGE, MA 02138

Dear Editors,

We would like to submit our revised manuscript, “General object-based features account for letter perception”.

There were two major comments, which required training new models and conducting new analysis.

First, Reviewer 2 again advocated for training models with letters depicted on scene backgrounds. We trained these models and have included these results.

Second, Reviewers 2 and 3 requested follow up explorations about the representational structure of the letter-trained network. To address these comments, we trained multiple iterations of AlexNet on letter classification to ensure the consistency of our results and reduce the presence of outliers in the representational structure, and we included new analyses comparing it to a purely categorical representational space.

We believe including these analyses in the updated manuscript has increased its clarity. We look forward to hearing from you about whether this work is acceptable for publication. A point-by-point response to reviewer comments is below.

Please don’t hesitate to contact us if you have any further questions. Thank you for your consideration.

Sincerely,

Daniel Janini, Chris Hamblin, Arturo Deza, and Talia Konkle

**Reviewer #1**

The authors have done a great job at addressing my concerns, congratulations on a revision well done.

The paper can likely be published as is, but I would like to take this opportunity to, nevertheless, highlight one subtle remaining concern about the take home message provided, for example in the author summary. To me, the paper shows nicely that letter perception can be well explained by more general visual features useful for object recognition, rather than requiring extensive training on domain-specific letter data. This, however, does not directly speak to the question which features humans use to recognize/classify letters while reading. Put differently, classification of letters into different categories may rely on different mechanisms than judging their visual similarity (which could be the implicit task studied here given the experimental setup). Maybe this subtlety could be acknowledged more explicitly. A minor point, I hope the authors forgive my pedantry.

- **Thank you for this point! We have made a few changes in the manuscript to increase clarity. We have edited the author summary to avoid implying that we directly address how letters are perceived in the context of reading. We have also added content to the discussion section to clarify that further research is needed to determine whether our findings generalize to the context of reading.**

**­**

**Reviewer #2**

I want to thank the authors for their careful consideration of my comments on the previous version of the manuscript. They have carried out many of the analyzes I suggested, along with changes made based on feedback from the other reviewers. I am sure it was quite a bit of work to make these revisions. Overall, I think the manuscript is substantially improved.

I still have two remaining concerns, though I am confident that the authors can address them.

Major comments:

A. In the previous round I worried that the study involved comparing “apples to oranges” because the letter-trained network only sees letters varying in font. Based on my recommendation, they added many degrees of variation (size, color, noise, etc.) and still found the same results. That was great to see (both that the analyses were done and that the results were the same!). However, the real underlying concern was that the letters were presented without natural scene information. Here the authors made the case for not including this analysis, because perhaps the network would learn to recognize objects to boost recognizing letters, for example.

However, I am afraid I am not convinced, but perhaps I wasn’t clear enough about what I had in mind. Whether showing photos of letters on surfaces in natural images might create problems, most simply scenes could be included in a counterbalanced way as used extensively in the work out of James DiCarlo’s lab. In that group’s stimuli (which I am confident the authors are aware of), different rendered objects are presented on random scene backgrounds. There is no relationship between scene and object type, so (for example) a DNN trained on their images does not learn to recognize scenes in order to categorize objects. But it nonetheless must learn to recognize objects in the presence of scene information. Doing something like this for letters would suffice to address my apples-to-oranges concern. Even like the DiCarlo group, you could even show the scenes in grey scale, and randomly present them behind the letters. This would rule out that it is the presence of natural scene information that explaines the difference, and would strengthen the case that it is something about representing object properties that explains the superior correspondence for the object trained model.

- **We have now trained a network to classify letters with scene backgrounds. To train this network we collected a set of 7,071 scene images from the SUN database. Scene images were excluded which had clear text. These scene images were randomly included as backgrounds during training. We have added results from this model to the manuscript in the “Additional Model Comparisons” section, and we have added details on how this model was trained to the methods section.**
- **Model-behavior correlations for this model can be viewed below. The letter-network trained with scene backgrounds never exhibited significantly higher model-behavior correlations than object-classifying AlexNet. Moreover, the model-behavior correlations for the final layers were still very low. This indicates that the network drives toward a representational space very different from human perception. In contrast, the object-trained network exhibits high model-behavior correlations from middle layers through the final layers.**

- **Including scene backgrounds did have a moderate effect on the representational structure of the letter-trained network. Below is a figure showing the correlations between the letter RDMs from the letter-networks trained with and without scene backgrounds.**

B. Per my suggestion, the authors carried out further analyses looking at the correlation with a pixel model and an un-trained model. The pixel results are plotted in Figures 1 B-C, and show an equivalent effect for the object and letter trained models through the first two conv layers, rho = ~ 0.4. For the object trained network the correlations increase at the third layer and basically stay at the same level. In contrast, for the letter-trained model correlations rise for both tasks at layer 4, and then start to freefall reaching 0 for the fc layers for the visual search RDM and rho = ~ 0.2 for the letter categorization task.

It was not salient until having the pixel model as a benchmark, but it seems like something strange is going on here with the letter trained model. It would be one thing if the correlations remained above the pixel correlation level through layers 5-7, but were simply lower than the object trained network. But this seems to show that the fc layers have learned a representation that is almost entirely *unrelated* to the letter dissimilarities.

I am not sure what is going on with this. Minimally I think the authors need to discuss why this might be the case and the implication for interpreting the results (I looked for a place where this was already done in the manuscript. If I missed it, I apologize). For my part, it makes the story less about how much better the object model does and rather how poorly the letter model does, which is not quite as compelling. My instinct though is that something weird has happened with the training of the letter model, but I am not sure what.

Just a hunch, but I wonder whether this drop in correlation at final conv and fc layers would happen if the authors took up my suggestion from comment A and trained the letter networks with letters presented on random natural scene backgrounds. If the correlations stayed above the pixel level in that case for the letter trained model through the later layers, I think that would substantially strengthen the results. But even if there is still a drop, it would at least rule out the possibility that it has something to do with the model not being exposed to natural image properties.

I also wonder if the drop in correlations is somehow related to the issue raised by Reviewer 1 about the letter trained model being optimized for discriminating letters, not representing their similarity. But that is just speculation on my part.

- **In the updated manuscript we add an analysis exploring why the letter-trained network diverges from human perception in the later layers. More specifically, we explore the possibility that the letter-trained network is too optimized for letter classification and doesn’t have graded similarity between letters in the later layers. These results can be found at the end of the Results section “Letter Categorization”**
- **Specifically, we created a model RDM which only represents the identities of letters and is abstracted from a perceptual similarity space. The fully connected layers of the letter-trained network exhibited low correlations with this identity-based RDM (r = 0.30, and r = 0.26, for Layers 6 and 7, respectively). Thus, the letter-trained model does have a graded similarity structure for letters in the final layers, it just doesn’t correspond with human letter perception.**
- **Additionally. as noted above, the letter-network trained with scene backgrounds also showed a drop in model-behavior correlations in the later layers.**

Minor comment:

In the new added text (blue) I noticed p-values are sometimes reported without the effect size of the accompanying measure. I think it would be helpful to say what the rho value was in the text in those cases.

- **In this paper we often summarize sets of seven p-values conducted for each layer of our neural networks. Reporting the corresponding rho values for all statistical tests would make the paper very hard to read. Instead, we have opted for putting all model-behavior correlations in a table in the Supplementary section.**

**Reviewer #3**

The authors have provided an extensive revision with new simulations, expanded stimulus sets for training, and important clarifications to the main text. Overall, I remain quite positive about the manuscript. These new analyses and revisions have strengthened the main claims, though also raise several new concerns that need to be resolved.

- **Thank you for the positive feedback!**

Looking at the RDMs in Supplementary Figure 2, the similarity structure for the updated letter-trained AlexNet looks odd in the deeper layers. It seems likely that the main reason for the poor match to behavior is due to the substantial distance of letters k and m from the rest of the letters. It is not clear why those letters would emerge to be so dissimilar in their activation patterns in comparison to all other letters. Perhaps this anomaly is masking a good correspondence with behavior for all other letters. This distinction of k and m from the rest of the letters was not present in the prior version of the model and needs to be resolved.

- **Starting from different random seeds, we have trained five more iterations of letter-trained AlexNet to determine if k and m are consistently outliers in the representational spaces of the later layers. Below we show MDS plots of Layers 6 and 7 in each network iteration. The letters k and m consistently stood out, though the extent to which this is true varied across training initializations. In the previous draft of the paper, we happened to use a network iteration that particularly made k and m outliers.**

- **In the revised manuscript, we have now included a letter-trained model with more typical representational structure. To select this model, first, we computed the mean layer-wise RDMs across the network iterations. Second, for each network iteration we computed the Spearman correlation between its layerwise RDMs and the mean layerwise RDMs. Finally, we picked the model with the highest correlations between its layerwise RDMs and the mean layerwise RDMs. This model does not show such excessive outliers as in the previous draft. All of the main results have been updated to include this network, and the methods section now includes information about this process.**
- **Regarding the main hypotheses tested in this study, our choice of which network iteration to include in the paper did not change the pattern of results. All letter-trained network iterations exhibited lower model-behavior correlations than object-trained AlexNet. In addition, all network iterations exhibited model-behavior correlations near 0 in the fully connected layers. However, some differences arose in comparison to pixelwise and random feature models. Previously, one layer of the letter-trained model exhibited higher model-behavior correlations than the pixelwise and random feature models, whereas in the updated draft the letter-trained network did not consistently exceed the pixelwise and random feature models.**
- **We would also like to clarify that we are comparing the model RDM and behaviorally measured RDMs with Spearman correlation instead of Pearson correlation, so outliers will not excessively reduce model-behavior correlations if the representational structure for the other letters corresponds with behavior.**

What was the motivation for setting the shallower network's depth to be layer 5? Looking at results from the AlexNet in the main analyses, model-behavior correlations were strongest at layer 4 then took a nosedive. From that, wouldn't a shallow network capped to the layer 4 make more sense (ie only layers that showed good correlation with behavior)? In the prior version of the manuscript, the shallow network was set to a depth of layer 4. It's unclear why the authors added an extra layer in the revision.

- **We set the smaller network’s depth to five layers because we found that such an architecture could learn to classify letters across the expanded image set and additional augmentations used in the updated manuscript.**
- **The smaller network not only has fewer layers than AlexNet, but it also has many fewer features per layer than AlexNet. Given these differences, it is difficult to directly compare layers of similar depth between the two networks. For example, in the first three convolutional layers, the smaller network had 40, 20, and 60 features, while AlexNet has 64, 192, and 384 features. Because of these differences, we do not believe one can infer that the shallower network should have been limited to four layers.**
- **We have edited the wording of the results section to clarify that the smaller network differed from AlexNet in both depth and numbers of features.**

The authors make an interesting prediction that letter learning will not create new patterns of responses to letters in the VWFA, but rather will increase the amplitude of those patterns. I struggled with this distinction. Wouldn't increasing the amplitude of responses to letters change the patterns - i.e. result in a different similarity structure? This claim needs to be unpacked. It's unclear how this prediction would manifest in the models tested in the manuscript.

- **Thank you for your question. We have edited the discussion section to clarify this point.**

The additional model comparisons (eg pixelwise comparisons and stylized AlexNet) are all nice additions, but need better motivated in the results to explain how these serve as tests of robustness. For example, the use of a shallower network in the following paragraph was well motivated in the following paragraph.

- **Thanks for pointing this out. We have edited the Results section “Additional Model Comparisons” to more clearly state the relevance of the pixelwise model, untrained AlexNet, and stylized-AlexNet.**
